# Diagnostic Performance of US and MRI in Predicting Malignancy of Soft Tissue Masses: Using a Scoring System

**DOI:** 10.3389/fonc.2022.853232

**Published:** 2022-04-29

**Authors:** Hua Shu, Qian Ma, Ao Li, Pingping Wang, Yingqian Gao, Qiyu Yao, Yu Hu, Xinhua Ye

**Affiliations:** ^1^ Department of Ultrasound, The First Affiliated Hospital of Nanjing Medical University, Nanjing, China; ^2^ Department of Ultrasound, Affiliated Zhongda Hospital of Southeast University, Nanjing, China

**Keywords:** soft tissue mass, ultrasonography, magnetic resonance imaging, neoplasm, diagnosis

## Abstract

**Objective:**

To assess the diagnostic performance of US and MRI in predicting malignancy of soft tissue masses by using a scoring system.

**Methods:**

A total of 120 cases of pathologically confirmed soft tissue masses (71 cases of malignant lesions and 49 cases of benign lesions) were enrolled. All patients underwent ultrasound and MRI examination prior to biopsy or surgical excision. A scoring system based on the parameters of conventional US and MRI to distinguish malignant and benign masses was established by the regression model. The receiver operating characteristic (ROC) analysis was used to evaluate the diagnostic performance of US and MRI.

**Results:**

Multivariate analysis showed that margin, maximum diameter, and vascular density were independent predictors for malignancy found by US, while maximum diameter, margin, and affected peripheral soft tissue were independent predictors for malignancy found by MRI. The mean scores of the benign and malignant groups were 2.8 ± 1.6, 5.1 ± 1.1 on US and 1.3 ± 1.2, 3.5 ± 0.9 on MRI. Based on the cut-off score of 3.5 and 2.5 calculated by ROC analysis, US and MRI had 92% and 87% sensitivity, 72% and 76% specificity, 86% and 89% accuracy, respectively. The combination of these two modalities achieved the sensitivity of 91%, specificity of 82%, and accuracy of 93%.

**Conclusions:**

Both US and MRI can provide valuable information about the differential diagnosis between benign and malignant soft tissue masses. The combination of the two imaging-based scoring systems can increase the diagnostic performance, especially in specificity.

## Introduction

Soft tissue sarcomas (STSs) are aggressive mesenchymal tumors consisting of more than 75 different histopathological subtypes. Due to the rarity and diversity subtype of STSs, it is difficult to diagnose accurately, and the clinical treatment is limited in lack of large-scale data guidance (1, 2). Despite the progress of treatment, the prognosis of STSs with metastasis or high grade is poor (3, 4). The pathological finding is still the standard differential diagnosis of benign and malignant soft tissue tumors (STTs). However, routine biopsy for each lesion which is suspicious of malignancy is not practical, and inadequate procedure would lead to a worse outcome in malignancy, in which improper placement and contamination of biopsy incisions could hinder limb salvage (5). Proper imaging examination can be helpful to narrow the scope of differential diagnosis and is crucial to guide further referrals (6).

Ultrasound (US) is the initial investigation in the evaluation of soft tissue masses due to its wide applicability, high sensitivity, nonionizing radiation, and low cost (7–9). US can provide information on the size and anatomical location of the lesions and can easily distinguish solid and cystic lesions (10, 11). Moreover, with the use of color Doppler, US can also reveal the hemodynamic changes within the lesions (7, 12–14). MRI is considered the initial investigation of choice for large deep lesions for localization, characterization, and staging (15). It is well-suited for evaluating local staging and assessing the anatomic extent of STTs because of its high intrinsic contrast resolution (16–18). Previous studies have reported that lesion characteristics, such as necrosis, fascial edema, signal heterogeneity on T_1_- and T_2_-weighted imaging (WI), deep localization (16, 19–21), and tumor-fascia relationship (21, 22) were useful in differentiating benign and malignant masses. Imaging examinations can evaluate the nature of the mass, as well as improve the level of experience of the musculoskeletal radiologist, to some extent (9, 23). Providing a confident diagnosis of tumor types or determining the likelihood of malignancy over image-based scoring system has considerable clinical benefits, which may assist clinical decision making. However, the value of these US and MRI parameters to characterize soft tissue masses remains controversial and no consensus feature can be used directly to distinguish malignant from benign soft tissue masses accurately (24, 25).

In the present study, we sought to develop a practical scoring system based on B-mode US and MRI parameters for helping discriminate malignant soft tissue masses from benign lesions.

## Materials and Methods

### Study Participants

This retrospective research was carried out according to the ethical standards of the Declaration of Helsinki and was approved by the ethics committee of the First Affiliated Hospital of Nanjing Medical University. The participant’s privacy and personally identifiable information is protected. Inclusion criteria were as follows: cases with pathological diagnosis of soft tissue masses in the authors’ institutions between January 2018 and May 2021, application of conventional US examination and MRI before surgical treatment or biopsy and availability of clinical, pathological, and radiological data. Exclusion criteria were cases with previous treatment such as biopsy, surgical excision, chemotherapy, or radiotherapy.

Ultimately, a total of 120 patients with soft tissue masses were enrolled. The following clinical characteristics including age, gender, course, histologic type of tumor, and anatomical site of the lesion were obtained from the medical records.

### Ultrasonography Examination

US examination was performed using a GE Logiq E9 US scanner (GE Healthcare, Milwaukee, WI) with the linear (6-15 MHz) and convex (2-6 MHz) transducers. The B-mode image had been determined to include the target lesion for the optimum resolution. The size of the sampling frame was adjusted to completely envelop the mass in color Doppler ultrasound mode. The color gain had been adjusted to a level that could detect low-velocity vascular flow in the target lesion with minimal background noise. The velocity scale of color Doppler examination was 6 cm/s. Multi-section scanning was adopted to reveal the maximum amount of vascular flow at the target lesion. All images were reviewed by two trained radiologists who had 3 and 6 years of experience in musculoskeletal ultrasound, respectively. If their diagnosis was inconsistent, the images were judged again by a chief physician with 10 years of experience in musculoskeletal ultrasound.

The following characteristics were assessed and recorded by the grayscale US: (1) layer (superficial/deep: relative to the investing fascia); (2) maximum diameter; (3) shape (regular, lobulated or unregular); (4) margin [smooth, partial unsmooth, or unsmooth (angular, or microlobulated)]; (5) boundary [well-defined, partial ill-defined, or ill-defined (uncertain boundary with respect to adjacent normal tissue)]; (6) echogenicity (hypoechoic/hyperechoic/isoechoic: relative to adjacent muscle tissue); (7) internal composition (solid, cystic-solid mixed, or cystic); (8) internal texture (homogeneous or heterogeneous); (9) calcification [(microcalcifications (punctate echogenic foci of less than about 1 mm with or without shadowing), macrocalcifications (echogenic foci that are larger than 1 mm, usually accompanied by posterior shadowing) or no calcification)]; (10) peripheral soft tissue (echo change in the soft tissue around the mass or no change), and (11) bone destruction (Y/N: continuity of cortical bone).

Color Doppler was used to evaluate tumor vascularity: (12) the vascular density was graded according to the semi-quantitative method as follow: no obvious blood flow in the mass (type I); only minimal blood flow, such as 1 to 2 punctate or rod-shaped blood flows in the mass (type II); moderate vascularity, such as 3 to 4 punctate blood flows or an important blood vessel which can be detected in the mass (type III); marked vascularity, such as more than 4 blood vessels or vessels are interwoven into a network(type IV); (13) vascularity patterns were based on Giovagnorio’s criteria: vascularity pattern was coded as avascular (type I), hypovascular with vascular pole in the hilum (type II), hypervascular with internal vessels (type III), or hypervascular with peripheral poles and hypervascular with internal vessels (type IV).

### MRI Protocol

Magnetic resonance imaging was performed using a 3.0 T MRI system (Siemens Magnetom Avanto, German). Conventional MRI protocols included axial and coronal T_1_WI, axial and sagittal fat suppressed T_2_WI. Another two trained musculoskeletal radiologists (with 3 and 5 years of experience) evaluated and recorded the following MRI parameters: maximum diameter, layer, signal intensity, texture pattern, internal composition, shape, boundary, margin, calcification, bone destruction, peripheral soft tissue, and location. Scorers were blinded to any other imaging performed before. If their diagnosis were inconsistent, the images were judged again by a chief physician with 10 years of experience in musculoskeletal radiology.

Most of the parameters were determined based on the fat suppressed T_2_WI, but the internal composition was combined with T_1_WI. Signal intensity (SI) was defined as homogeneous high SI (type I), homogeneous low SI (type II), heterogeneous SI with less than 50% low SI in high SI (type III), and heterogeneous SI with over 50% low SI in high SI (type IV). The other observational factors referred to the parameters of grayscale US.

### Statistical Analysis

In the univariate analysis of training cohort, categorical data were compared with the chi-square test or Fisher’s exact test, and continuous data were compared with Mann-Whitney U-tests to obtain independent risk factors for malignant soft tissue masses. P<0.05 was considered statistically significant. The cutoff values were assessed by receiver operating characteristic (ROC) analyses with the significant factors as continuous variables.

Before multivariable analysis, this article made a further analysis of parameters of clinic, US, and MRI, using Spearman’s correlation coefficients. Variables with statistical significance in univariate analysis and good correlation with pathological results were input into the multivariable analysis by the binary logistic regression model. Finally, according to the odds ratio acquired by the regression model, the ideal combined weight of each parameter was calculated. The sum of scores in each category was analyzed by ROC analysis. The area under the ROC curve between groups was compared using the Delong test. Statistical analyses were performed with statistical software (SPSS, version 25.0, SPSS).

## Results

### Clinical Features

The clinical characteristics of patients and the pathological categorization are summarized in **Table 1**. The final participant included 58 male and 62 female with average age of 51.8 ± 15.6 years. There were 49 benign and 71 malignant lesions consisting of more than 30 different tumor types (**Table 1**). The gender, age, and course proved to be different between benign and malignant masses (P<0.005). In addition, the cutoff values of 47.5 years of the age and 10.5 weeks of the course were determined, achieving a diagnostic accuracy of 0.65 and 0.61, respectively.

**Table 1:** Pathological diagnosis of the patients in the present study.

	Malignant (n = 71)	Benign (n = 49)	Total (n = 120)
**Sex**			
** Male**	41	11	58
** Female**	30	38	62
**Mean Age (years)**	55.2	46.9	51.8
**Course (weeks)**	26.8	6.5	14.8
**Location**			
** Upper extremity**	10	9	19
** Lower extremity**	42	29	71
** Trunk**	19	11	30
**Pathology**	Myxofibrosarcoma (n=4)	Fibromatosis (N=6)	
	Pleomorphic sarcoma (n=5)	Fibroma (N=3)	
	Liposarcoma (n=8)	Neurinoma (N=9)	
	Synoviosarcoma (n=5)	Lipoma (N=9)	
	Solitary fibrous tumor (n=4)	Hemangioma (N=5)	
	Rhabdomyosarcoma (n=1)	Giant cell tumor of tendon sheath (N=4)	
	Leiomyosarcoma (n=2)	Mixed tumor (N=3)	
	Chondrosarcoma (n=3)	Granulomatous inflammation (N=4)	
	Malignant peripheral nerve sheath tumor (n=2)	Baker’s cysts (n=1)	
	Metastasis of malignant tumor (n=15)	Other (n=5)	
	Lymphoma (n=6)		
	Other (n=16)		

### US Characteristics

After investigation of all US variables of soft tissue masses, univariate analysis showed that significant associations were observed between malignancy and maximum diameter (P<0.001), shape (P<0.001), boundary (P=0.003), margin (P<0.001), bone destruction (P=0.005), vascular density (P<0.001), vascularity patterns (P<0.001), and echogenicity (P<0.001). In addition, the cutoff value of 50.5 mm of the maximum diameter was determined, achieving a sensitivity of 83% and a specificity of 65%. The area under the ROC curve (AUC) (0.80) suggested that the cutoff value had favorable effective functions for the diagnosis of malignancy.

**Figure 1:**
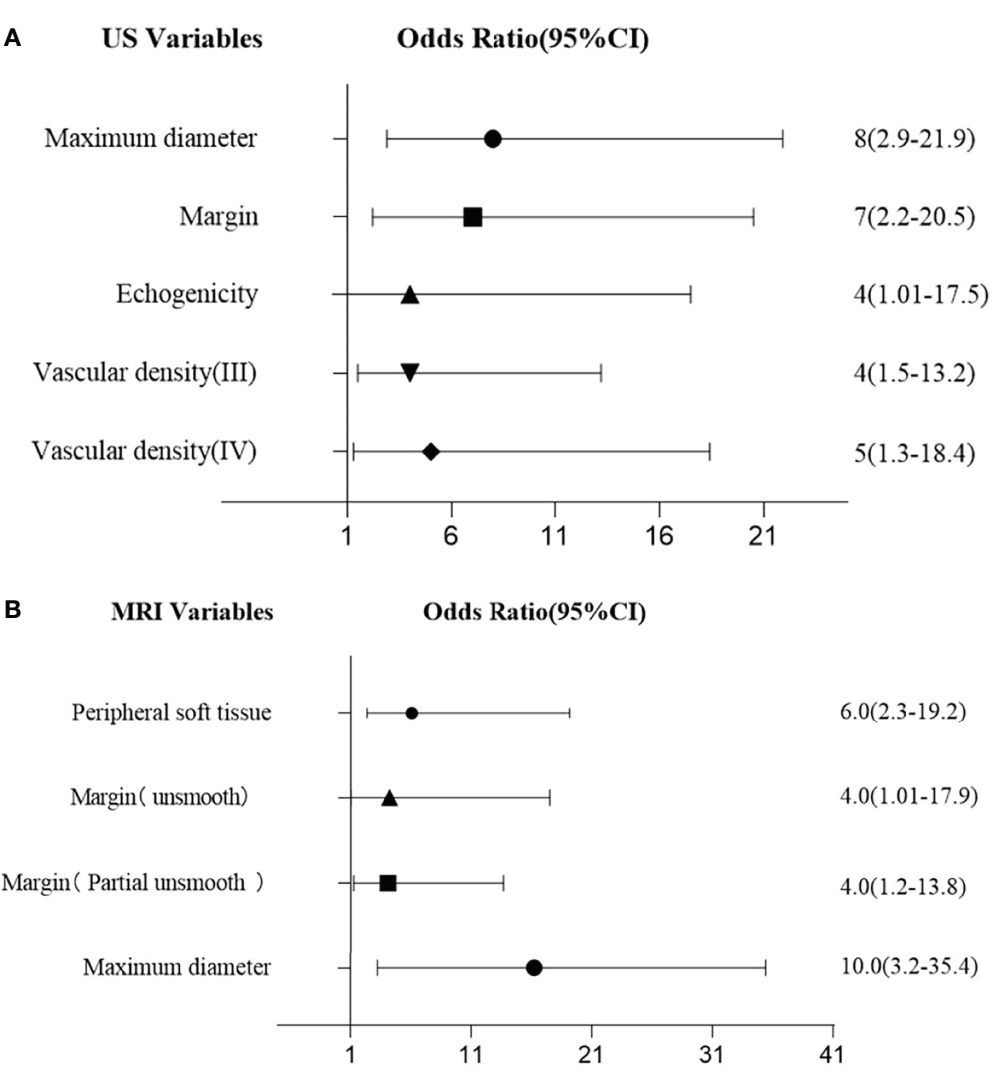
**(A)** Multivariate logistic regression for US features. **(B)** Multivariate logistic regression for MRI features.

From the multi-variate analysis, independent factors of soft tissue masses included margin, maximum diameter, echogenicity, and vascular densities (**Table 2**) (**Figure 1**).

**Table 2:** Multivariate logistic regression model with odds ratios composed of independent factors in US.

Parameters	Odds ratio	Score	95%CI	P
Maximum diameter				<0.05
Less than 50.5 mm	Reference	0		
Over 50.5 mm	8	2	2.9-21.9	0.001
Margin				<0.05
Smooth	Reference	0		
Partial/unsmooth	7	2	2.2-20.5	0.001
Echogenicity				<0.05
Hyperechoic/isoechoic	Reference	0		
Hypoechoic	4	1	1.01-17.5	0.048
Vascular density				<0.05
Type I/II	Reference	0		
Type III	4	1	1.5-13.2	0.008
Type IV	5	1	1.3-18.4	0.021

### MRI Findings

According to the univariate analysis of MRI findings, both maximum diameter, texture pattern, shape, boundary, margin, bone destruction, and peripheral soft tissue had significant statistical difference in differentiating between benign lesions and malignant (P<0.05). Cut-off value was 45.5 mm for maximum diameter of the tumors.

With multivariate analysis, maximum diameter, margin, and peripheral soft tissue were independent factors for differentiating benign and malignant soft tissue masses (**Table 3**) (**Figure 1**).

**Table 3:** Multivariate logistic regression model with odds ratios composed of independent factors in MRI.

Parameters	Odds ratio	Score	95%CI	P
Maximum diameter				<0.05
Less than 45.5 mm	Reference	0		
Over 45.5 mm	10	2	3.2-35.4	0.001
Margin				<0.05
Smooth	Reference	0		
Partial unsmooth	4	1	1.2-13.8	0.018
Unsmooth	4	1	1.01-17.9	0.049
Peripheral soft tissue				<0.05
No	Reference	0		
Change	6	1	2.3-19.2	0.001

### Diagnostic Performance of Conventional US and MRI in Benign and Malignant STT Group

**Figure 2:**
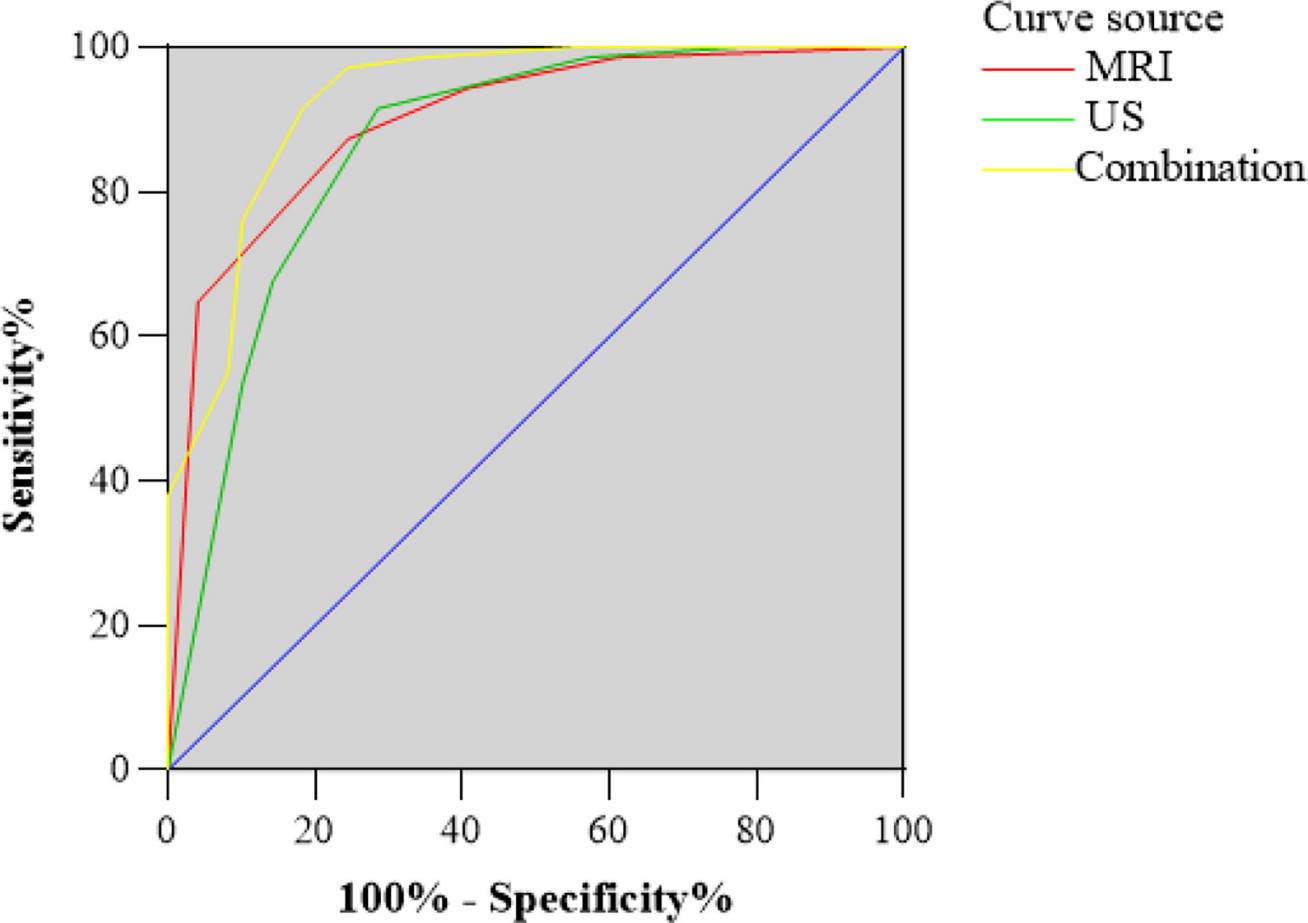
Receiver operating characteristic (ROC) curve for US and MRI classification of the total scores. The area under the curve (AUC) was 0.86 and 0.89, respectively. ROC curve for the combination of US and MRI classification of the total scores. The AUC was 0.93.

**Figure 3:**
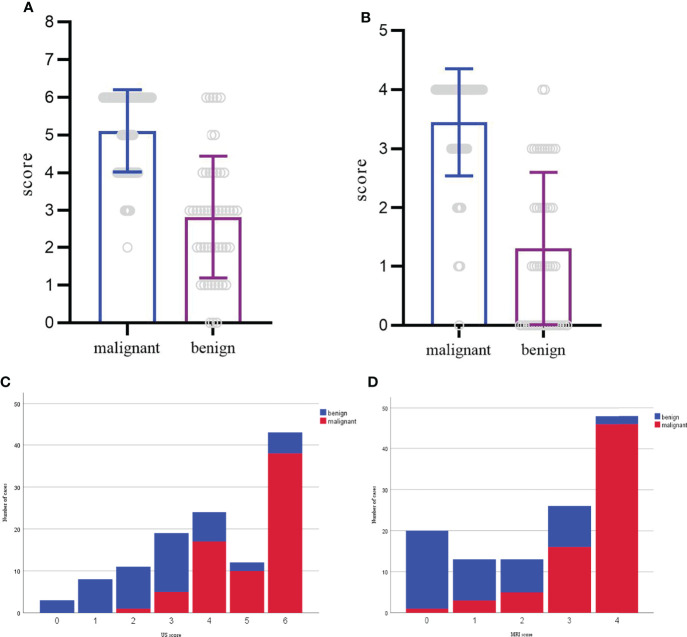
**(A, C)** Distribution of benign and malignant cases according to the US scoring system. The score of each mass ranged from 0-6. **(B, D)** Distribution of benign and malignant cases according to the MRI scoring system. The score of each mass ranged from 0-4.

Based on these odds ratios from the multivariate logistic regression, a scoring system was developed. The final scores of soft tissue masses were acquired by adding up the scores of each indicator. The final scores of the benign and malignant groups were 2.8 ± 1.6, 5.1 ± 1.1 in the US (P<0.05). With the cutoff value of 3.5, the corresponding US scoring system gave a sensitivity of 0.92, a specificity of 0.72, and an area of ROC curve of 0.86. The final scores of the benign and malignant groups were 1.3 ± 1.2, 3.4 ± 0.9 in MRI (P<0.05). With the cutoff value of 2.5, MRI scoring system showed the sensitivity, specificity, and accuracy were 0.87, 0.76, and 0.89 (**Figures 2**, **3**). Although US had a higher sensitivity, the difference of AUC was not statistically significant between US and MRI (P=0.71).

The imaging-based scoring system combined with US and MRI showed a sensitivity of 0.91, a specificity of 0.82, and diagnostic accuracy of 0.93 (**Figure 2**). In terms of AUC, the combination of US and MRI performed better than MRI alone in differentiating between benign lesions and malignant (P=0.04).

## Discussion

This study showed that the novel scoring system based on conventional US and MRI examination was helpful for the differential diagnosis of soft tissue masses. These findings supported US as an initial examination for soft tissue masses. While the two modalities were combined, the diagnostic ability of imaging-based classification could be effectively improved.

**Figure 4:**
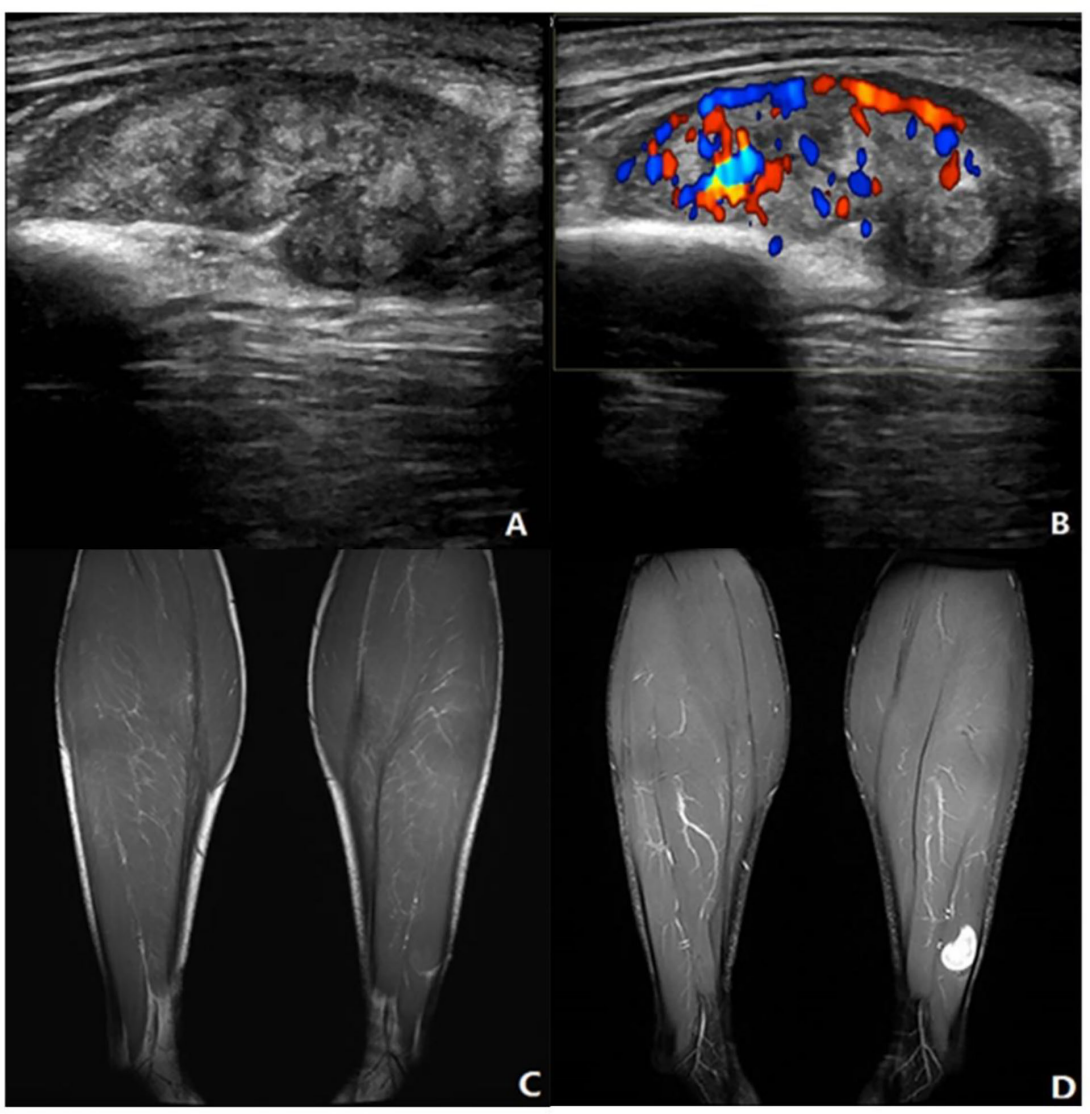
Neurinoma of lower extremity. A 41-year-old male presented with a mass in the left lower leg. **(A)** Longitudinal grayscale US showed a well-defined and heterogenous mass with maximum diameter of 34 mm in the peroneal brevis muscle. **(B)** Color Doppler imaging of the same area showed hypervascularity within the tumor, corresponding to type IV. **(C)** Coronal T1WI showed a homogeneous isointensity mass. **(D)** The lesion had homogeneous high SI on the coronal fat-suppressed T2WI with maximum diameter of 20 mm. **(A, B)** A score of 2 was assigned, indicating a benign tumor (true positive). **(C, D)** A score of 0 was assigned, indicating a benign tumor (true positive).

In previous studies, US features such as tumor size, vascularity, margin, and echogenicity were suggested to be useful in providing confidence in the possibility of malignancy rather than a benign tumor (13, 14). In our study, the multivariate analysis showed that margin, maximum diameter (>50.5mm), echogenicity, and vascular density were independent factors in differentiating malignancy by US. Among these parameters, maximum diameter and margin accounted for a relatively large proportion in the scoring system and maximum diameter was given the highest score both in current and previous studies (14, 26). A lesion with a diameter >5 cm is strongly suspected as malignance in clinical practice (27). Our study confirmed the finding and the cutoff value calculated by ROC analysis was similar to those used in conventional guidelines. Angiogenesis and proliferation are also universal features of malignant tumors, which can be appreciably and non-invasively detected by color Doppler (28, 29). Tumor vascularity with type III and IV were defined as significant prognostic factors in determining malignancies. The scoring system based on US parameters from the multivariate logistic regression had a sensitivity of 92%, a specificity of 72%, and an accuracy of 86% (**Figure 4**), with the cutoff value of 3.5. Morii et al. established a scoring system of US revealing 83% sensitivity, 73% specificity, and 85% diagnostic accuracy, with maximum size, margin, and vascularity extracted as significant risk factors (14). In their study, echogenicity was not a useful parameter for this distinction and common masses such as ganglion, Baker’s cyst, and metastatic soft tissue masses were excluded, which may affect the evaluation of parameters, because different subtypes of soft tissue masses have quite different tumor composition. The usefulness of echogenicity for determining malignancy is controversial, which is one of the significant factors in our study. Nagano and Morii also reported that low echogenicity was a significant characteristic of malignant or high-grade STSs (13, 30).

**Figure 5:**
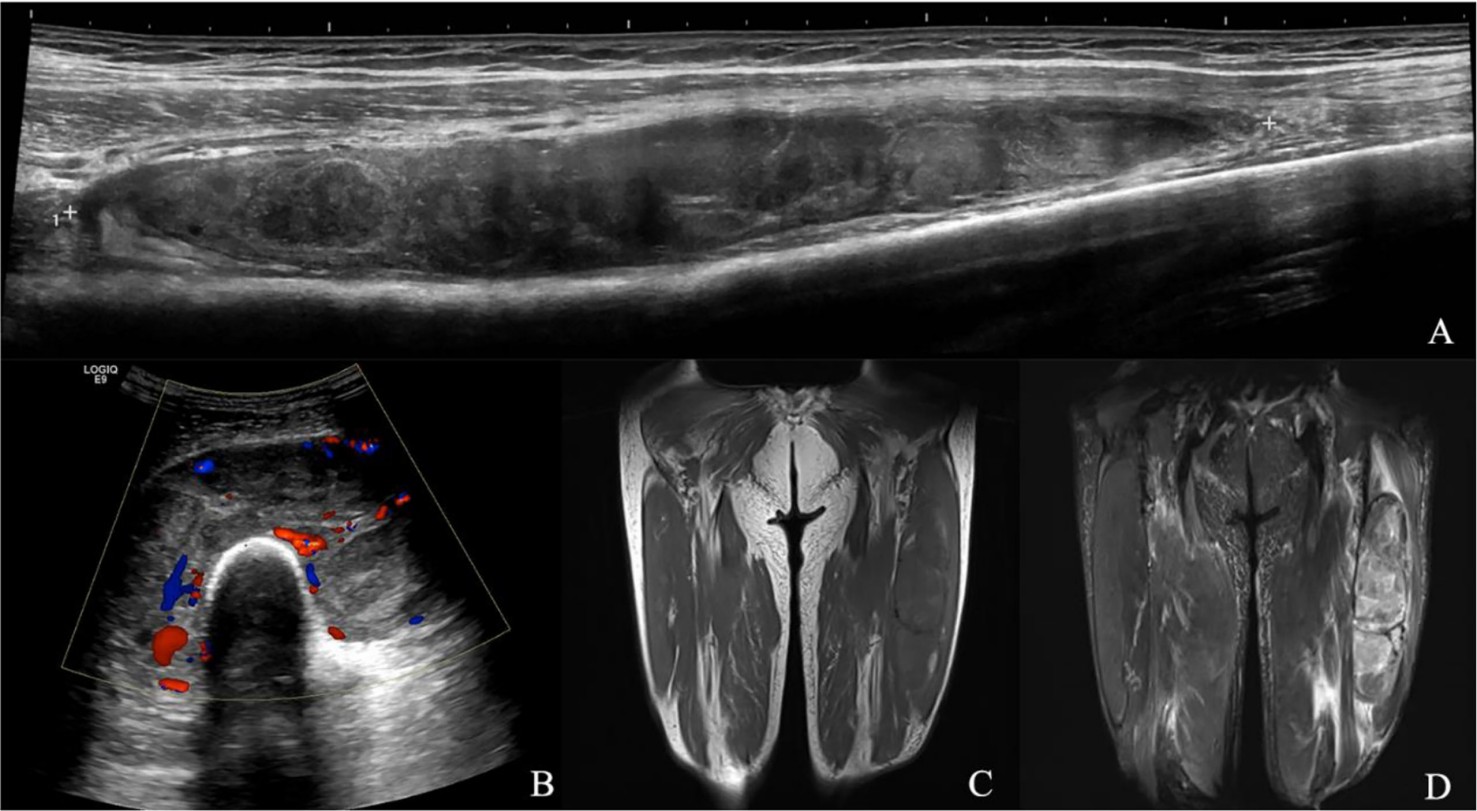
Myxofibrosarcoma of lower extremity. A 68-year-old male presented with a swelling in his left lower leg. **(A)** Longitudinal grayscale US showed a well-defined, partial unsmooth, heterogenous intramuscular mass with a maximum diameter of 201 mm in the intermediate vastus muscle. **(B)** Color Doppler imaging of the same area showed hypervascularity within the tumor, corresponding to type IV. **(C)** Coronal T_1_WI showed a heterogeneous intensity mass. **(D)** The lesion had relatively heterogeneous high SI on the coronal fat suppressed T_2_WI with maximum diameter of 278 mm and microlobulated margin. **(A, B)** A score of 6 was assigned, indicating a malignant tumor (true positive). **(C, D)** A score of 3 was assigned, indicating a malignant tumor (true positive).

MRI parameters including margin, maximum diameter (>45.5 mm), and affected peripheral soft tissue were independent factors from the multivariate analysis. The best predictor was the maximum diameter, which was consistent with the study of Winn et al. (26). The reactivity of the surrounding soft tissue changes was confirmed to be infiltrating viable cells or edematous change pathologically, which causes the seemingly appearance of unsmooth margin and peritumorous edema (31–33). With the cutoff value of 2.5, the MRI scoring model had a sensitivity, specificity, and accuracy of 87%, 76%, and 89%, respectively (**Figure 5**). Chung et al. assessed the systematic combination of signal intensity, size, and depth with a sensitivity of 64%, a specificity of 85%, and an accuracy of 77% (34). However, they only selected these three main parameters without evaluating other parameters. Likewise, they excluded patients with recurrent soft tissue masses or osteogenic lesions. It may be worth mentioning that comparison with previous findings is difficult as the spectrum of pathologies analyzed varies between studies.

In both US and MRI scoring system, tumor margin and maximum diameter seemed to contribute to the differential diagnosis of soft tissue masses, with higher scores than other parameters. Moreover, due to the different characteristics of these two imaging modalities, they can provide different information on tumors. US can reveal hemodynamic changes, whereas MRI can highlight abnormal changes and tissue edema. According to the scoring system, the diagnosing performance of US seemed similar to that of MRI, with a high sensitivity and a moderate specificity.

**Figure 6:**
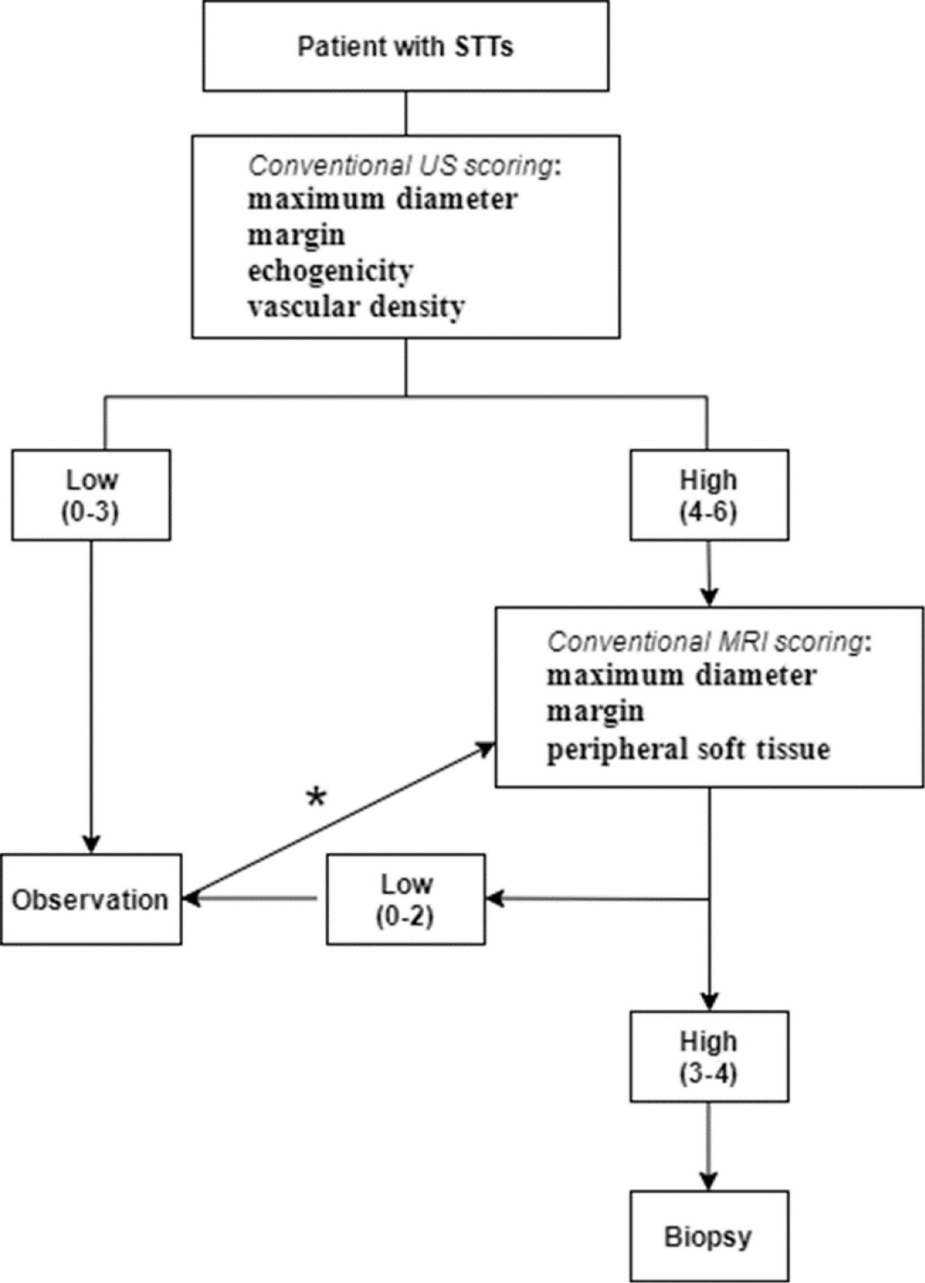
Workflow of the imaging diagnosis of STTs based on US and MRI scoring system. This flowchart presents a screening procedure for STTs. The US score is evaluated at the first visit. If the score is low (<3.5 points), the patient can be observed periodically over several months. If the tumor size increases rapidly or the score increases on the second visit or later, the physician may consider performing MRI (*). If the score is high at the initial visit (≥3.5 points), MRI may be recommended. When the MRI score is less than 3 points (<2.5 points), a short-term observation can be recommended, otherwise, it is suspected to be malignant (≥2.5points). Biopsy should be performed to obtain a pathological diagnosis for any mass considered suspicious of malignancy.

Our results implied that the imaging-based scoring system combined with US and MRI showed a better diagnostic ability and a more satisfactory specificity of classification compared with using US or MRI alone. However, Winn et al. found that the combination of US and MRI findings could not confidently distinguish a lesion from benign to malignant, and did not establish an evaluation method (26). Tavare et al. confirmed that the diagnostic accuracy was improved when US was combined with MRI, but they only concluded from the overall impression of the images (35). To generate a simplified systematic imaging-based scoring system, we added a more comprehensive assessment and a broader spectrum of disease in a large patient group. Furthermore, a workflow based on the scoring system for clinical decision was developed (**Figure 6**). US examination is recommended at the initial visit. If the imaging score is low (<3.5 points), the patient can be observed periodically over several months. But if the tumor size increases rapidly, the score increases on the second visit or later, or considering other suspicious tumor-related symptoms, MRI is recommended.

According to this scoring system, 6 malignant masses were misdiagnosed as benign lesions. Among them, 3 cases were recurrent sarcoma, which may be treated correctly through obtaining the information of clinical history. The other 3 misdiagnosed lesions include: malignant peripheral nerve sheath tumor (MPNST), myxoid liposarcoma (MLS), and myxoid fibrosarcoma (MFS). The detection of MPNST and its differentiation from benign neurofibromas (PNST) remains a clinical challenge, due to the similar symptomology including tumor size, pain, and neurologic deficits, as well as the definitive radiographic distinction (36). These data should be combined with a thorough history and physical examination or in conjunction with FDG-PET (36, 37). As histologic analysis of myxoid tumors reveals a myxoid matrix, conventional MRI may not be applicable to them (38). In accordance with prior literature reports, myxoid tumors represented the three out of four false-negative tumors in MRI analysis (19, 39). Advanced techniques may help define a clearer malignancy identification.

There are some limitations in the current study. First, as the deviation of common clinical decision, our final patients were mainly considered to have suspicious malignant or indeterminate lesions and underwent surgical excision or biopsy. Thus, there was a selection bias with a high portion of bigger lesions in this study. Further investigation is needed in expanding the selection criteria of the sample to evaluate the diagnosis priority including smaller lesions. Second, the lack of the assessment of interreader and intrareader reliability during investigation is also the limitation of this study, although readers were trained and had extensive experience. Third, only the conventional US and MRI characteristics were studied in this paper. However, some researchers found that elastography can provide valuable information about STTs (35, 40). Further efforts can use the advanced US and MRI images with elastography to evaluate their hopeful performances.

## Conclusion

In conclusion, we demonstrated the value of both US and MRI scoring system in malignancy prediction of soft tissue masses developed in this study. The US scoring system composed of several available parameters derived from conventional US could be a sensitive and noninvasive tool for the classification of soft tissue masses, especially for the primary screening. The combination of the two imaging-based scoring systems ultimately leads to improved overall diagnostic performance, but more importantly, it allows a clear management and minimizes the need for biopsies, unnecessary imaging, or follow-up.

## Data Availability Statement

The original contributions presented in the study are included in the article/**Supplementary Material**. Further inquiries can be directed to the corresponding authors.

## Author Contributions

Authors HS and QM wrote the first draft of the manuscript and performed the statistical analysis. AL organized the database. Authors YH and XY contributed to conception and design of the study. All authors contributed to manuscript revision, read, and approved the submitted version.

## Funding

This study has received funding by the National Natural Science Foundation of China (No. 81401427).

## Conflict of Interest

The authors declare that the research was conducted in the absence of any commercial or financial relationships that could be construed as a potential conflict of interest.

## Publisher’s Note

All claims expressed in this article are solely those of the authors and do not necessarily represent those of their affiliated organizations, or those of the publisher, the editors and the reviewers. Any product that may be evaluated in this article, or claim that may be made by its manufacturer, is not guaranteed or endorsed by the publisher.
